# Ultrasound-based radiomics for predicting different pathological subtypes of epithelial ovarian cancer before surgery

**DOI:** 10.1186/s12880-022-00879-2

**Published:** 2022-08-22

**Authors:** Zhi-Ping Tang, Zhen Ma, Yun He, Ruo-Chuan Liu, Bin-Bin Jin, Dong-Yue Wen, Rong Wen, Hai-Hui Yin, Cheng-Cheng Qiu, Rui-Zhi Gao, Yan Ma, Hong Yang

**Affiliations:** 1 Department of Medical Ultrasound, The First Affiliated Hospital of Guangxi Medical University, Nanning, Guangxi Zhuang Autonomous Region China; 2 Department of Medical Ultrasound, Guangxi International Zhuang Medical Hospital, Nanning, Guangxi Zhuang Autonomous Region China

**Keywords:** Epithelial ovarian cancer, Ovarian neoplasms, Histological classification, Ultrasonic examination, Radiomics

## Abstract

**Objective:**

To evaluate the value of ultrasound-based radiomics in the preoperative prediction of type I and type II epithelial ovarian cancer.

**Methods:**

A total of 154 patients with epithelial ovarian cancer were enrolled retrospectively. There were 102 unilateral lesions and 52 bilateral lesions among a total of 206 lesions. The data for the 206 lesions were randomly divided into a training set (53 type I + 71 type II) and a test set (36 type I + 46 type II) by random sampling. ITK-SNAP software was used to manually outline the boundary of the tumor, that is, the region of interest, and 4976 features were extracted. The quantitative expression values of the radiomics features were normalized by the Z-score method, and the 7 features with the most differences were screened by using the Lasso regression tenfold cross-validation method. The radiomics model was established by logistic regression. The training set was used to construct the model, and the test set was used to evaluate the predictive efficiency of the model. On the basis of multifactor logistic regression analysis, combined with the radiomics score of each patient, a comprehensive prediction model was established, the nomogram was drawn, and the prediction effect was evaluated by analyzing the area under the receiver operating characteristic curve (AUC), calibration curve and decision curve.

**Results:**

The AUCs of the training set and test set in the radiomics model and comprehensive model were 0.817 and 0.731 and 0.982 and 0.886, respectively. The calibration curve showed that the two models were in good agreement. The clinical decision curve showed that both methods had good clinical practicability.

**Conclusion:**

The radiomics model based on ultrasound images has a good predictive effect for the preoperative differential diagnosis of type I and type II epithelial ovarian cancer. The comprehensive model has higher prediction efficiency.

## Introduction

Ovarian cancer is the deadliest cancer of the female reproductive system [1, 2]. Approximately 13,940 women in the United States died of the disease in 2020 [3]. According to the female reproductive organ tumor classification system published by the World Health Organization in 2014 [4], type I epithelial ovarian cancer includes low-grade serous carcinoma, endometrioid carcinoma, clear cell carcinoma, mucinous carcinoma and malignant Brenner tumor. Type II epithelial ovarian cancer includes high-grade serous carcinoma, carcinosarcoma and undifferentiated carcinoma. Epithelial ovarian cancer has the highest fatality rate among malignant tumors of the female reproductive system, and different types are closely related to prognosis. The overall prognosis of type I is good, while that of type II is poor [5]. Therefore, it is of great clinical significance to improve the accuracy of preoperative diagnosis [3, 6]. Early identification of epithelial ovarian cancer subtypes is of great importance [7–9].

Traditional imaging is the main means of detecting ovarian tumors [7], but this method largely depends on the doctor's personal experience [10], and some tumor-specific imaging features cannot be recognized by the naked eye, resulting in clinical diagnosis inefficiencies. Radiomics is more objective than traditional imaging methods. It extracts high-throughput image features from traditional medical images to quantitatively analyze diseases and provides new insights into the clinical diagnosis and treatment of ovarian tumors [11, 12].

Radiomics, first proposed by Lambin et al. in 2012 [11], has developed rapidly in recent years [13]. It provides a noninvasive method for diagnosing and predicting diseases. Gulshan V et al. diagnosed diabetic retinopathy by analyzing 128,175 retinal images [14–16]. Yin et al. established a radiomics model using MR images and effectively identified chordomas, giant cell tumors and metastatic tumors before operation [17]. Peng et al. developed a radiomics model for the preoperative recognition of HCC and non-HCC using ultrasound images [18]. It is widely regarded as a step in the development of radiomics for personalized cancer management [13].

At present, there is a lack of ultrasound-based radiomics methods for distinguishing different subtypes of epithelial ovarian cancer before surgery [19]; therefore, the purpose of this study was to establish and verify an objective ultrasound-based radiomics evaluation model for the preoperative prediction of type I and type II epithelial ovarian cancer. Accurate prediction of tissue classification of epithelial ovarian cancer before operation can provide more accurate treatment plan for clinic and better decision-making for patients.

## Materials and methods

### Research population

**Fig. 1:**
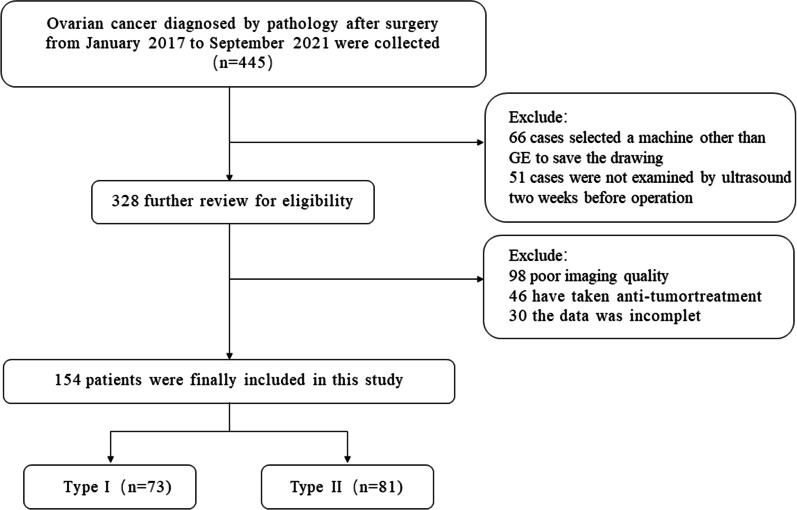
Flow chart of research population screening

The Ethics Committee of the First Affiliated Hospital of Guangxi Medical University approved this retrospective study (approval number: NO.2022 -KY-E-(056). Informed consent was waived. Patients with epithelial ovarian cancer diagnosed by pathology after surgery at the First Affiliated Hospital of Guangxi Medical University from January 2017 to September 2021 were enrolled (Fig. 1). The inclusion criteria were as follows: (1) primary epithelial ovarian cancer; (2) lesions confirmed by operation and pathology; (3) transvaginal ultrasound examination of ovaries within 14 days before operation; and (4) clear ultrasound images. The exclusion criteria were as follows: (1) preoperative anticancer therapy; (2) poor image quality; and (3) incomplete clinical data. Finally, a total of 154 patients were included, with an average age of 50.15 ± 10.80 years and a range of 21–76 years.

### Instruments and methods of ultrasonic examination

Using a GE Volusion E10 and E8 ultrasonic diagnostic apparatus, the transvaginal probe type was RIC5-9 Mel D, and the frequency was 5–9 MHz. Transvaginal ultrasonography was used to scan ovarian tumors from multiple sections and angles to understand the overall information. Then, we carefully scanned and observed the size, shape and echo of the tumor, selected the largest section of the tumor with the clearest imaging, and saved the image in medical digital imaging and communication format to maximize the preservation of the image information.

### Image segmentation and feature extraction

**Fig. 2:**
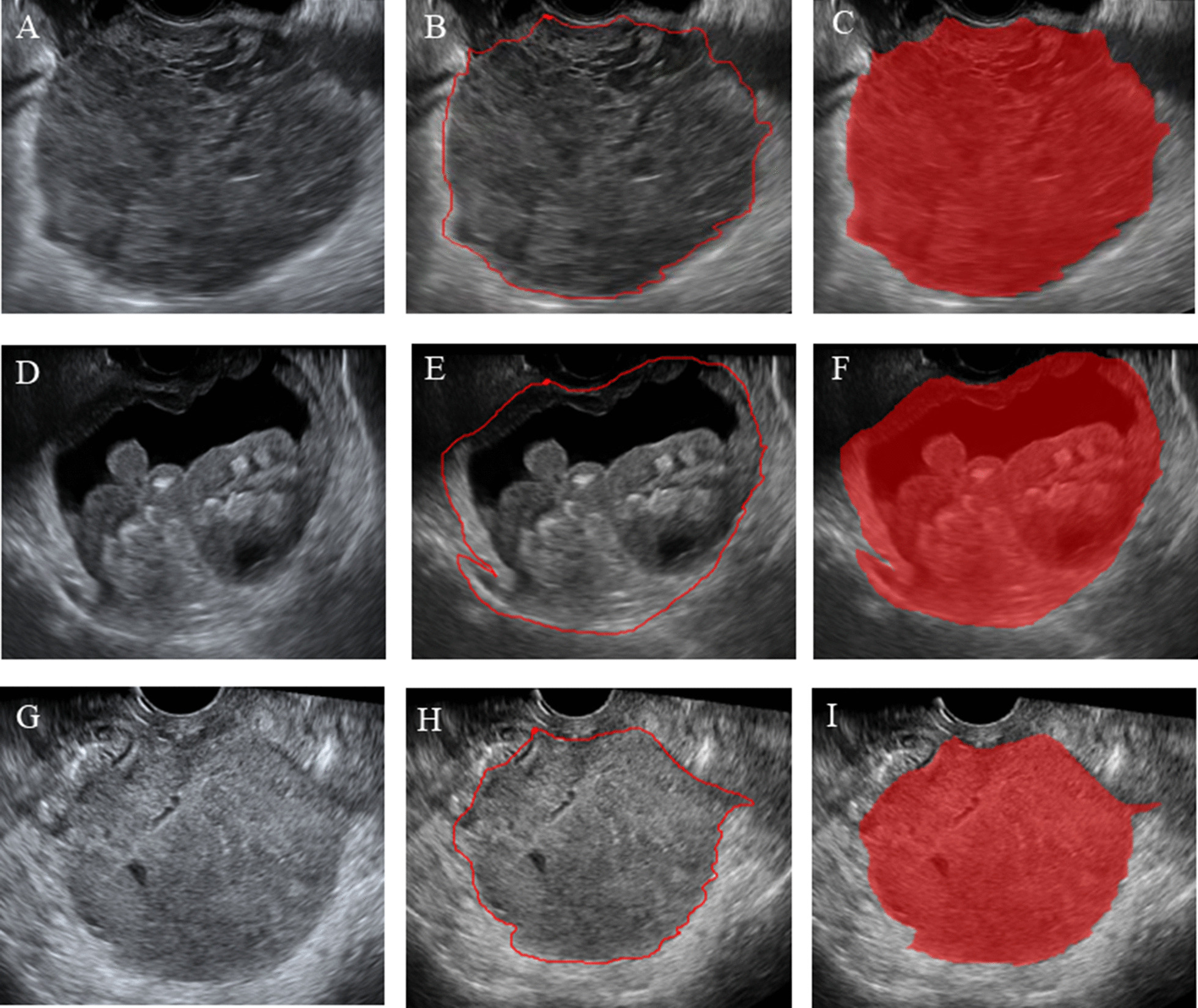
Schematic outline of the region of interest (ROI) of epithelial ovarian cancer.** A,D,G** The biggest section of the ultrasound image of epithelial ovarian cancer.** B, E, H** The red line delineates the ROI along the edge of the lesion.** C, F, I** Schematic of the cut image.** A, B, C** show a case of ovarian clear cell carcinoma.** D, E, F** show a case of low-grade serous carcinoma of the ovary.** G,H,I** show a case of high-grade serous carcinoma of the ovary

**Fig. 3:**
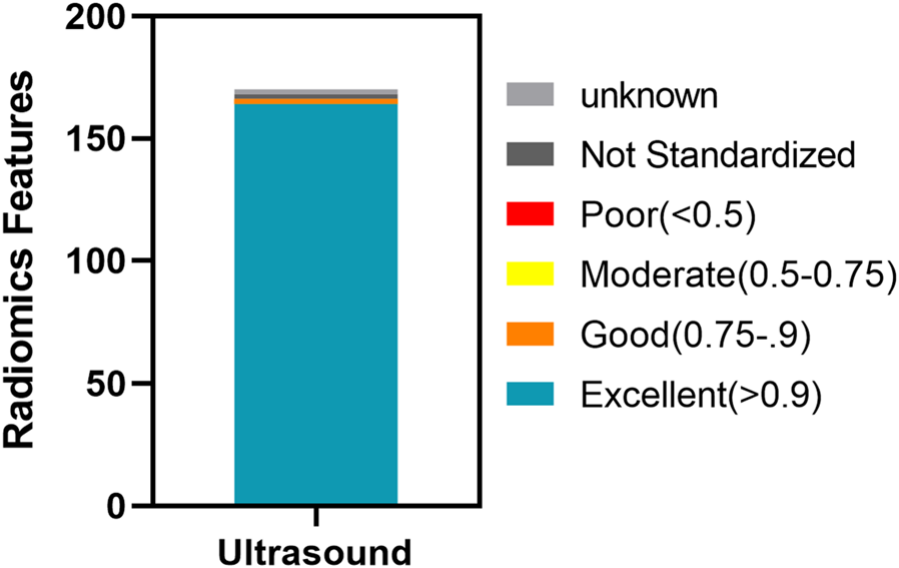
showed the repeatability of standardized imaging features through histograms. Using the lower bound of 95% confidence interval, the intra-class correlation coefficient of absolute consistency of features (difference < 0.50; moderate: 0.50–0.75; advantages: 0.75–0.90; excellent > 0.90). Figure 3 showed that the features extracted by the ROI sketched by the two doctors were highly repetitive

The image was imported into ITK-SNAP software (version 3.8) to manually draw the tumor boundary and determine the tumor area of interest (ROI) (Fig. 2). The ultrasonographic manifestations of ovarian cancer can be divided into two types, one is solid, the other is mixed. Our standard for drawing ROI is to draw along the edge of the tumor, if it is a solid mass, we will outline the whole solid part, if it is a mixed mass, we will outline the whole edge of the mass, including the solid part and the liquid part. All tumor areas of interest were delineated under the supervision of an ultrasound doctor with 10 years of ultrasound diagnosis experience and another ultrasound doctor with 15 years of experience in ultrasound diagnosis. Neither ultrasound doctor knew the pathology results. Also, 50 images were randomly selected from all the images and drawn independently by two doctors to evaluate consistency between different observers. 171features were selected from 50 images for repeated verification. Figure 3 showed the repeatability of standardized imaging features through histograms. Using the lower bound of 95% confidence interval, the intra-class correlation coefficient of absolute consistency of features (difference < 0.50; moderate: 0.50–0.75; advantages: 0.75–0.90; excellent > 0.90). The bar chart shows that the features extracted by the ROI sketched by the two doctors are highly repetitive.

IntelligenceFoundry software (GE Healthcare, version 1.3) was used to analyze and extract radiomics features. Feature types included first-order features (energy, mean, skewness, kurtosis, etc.), shape features (minor axis length, major axis length, extension, etc.), wavelet features and texture features [gray co-occurrence matrix (GLCM) features, gray run length matrix (GLRLM) features, etc.]. A total of 4976 high-throughput features were extracted in this study. The feature parameters extracted by the Intelligence Foundry software were based on the algorithm provided by the pyRadiomics package, which calculates radiomics features according to the feature definition described in the Image Biomarker Standardization Initiative (IBSI) version 2016 [20, 21]. The median was used to fill in the missing extracted eigenvalues and replace the outliers. The Z-score normalization method was used to convert different data into the same order of magnitude, and the calculation formula was as follows: y = (x μ)/σ, where μ is the mean and σ is the standard deviation.

### Data preprocessing

Patients with epithelial ovarian cancer were marked with different labels according to their histological types. Type I epithelial ovarian cancer was labeled "0", and type II epithelial ovarian cancer was labeled "1". Then, using the method of stratified sampling, patients with two histological types of epithelial ovarian cancer were randomly divided into two groups according to a 6:4 (training set:test set) ratio. The training set was used to build the model, and the test set was used to verify the effectiveness of the model.

### Ultrasonic parameters

In this study,all patients were diagnosed with GEVolusion ultrasound and were examined under the transvaginal probe.We collected the important ultrasonic parameters of all the pictures in this study, including gain,mechanical index,depth,angle. After analysis, we found that these important parameters were not statistically significant in the two types of ovarian cancer (P > 0.05). (Table 1).

**Table 1:** The ultrasound parameter of the type I and the type II are shown in

Variable	Type I (n = 89)	Type II (n = 117)	*P*
Gain (dB)	6.00 (2.00–10.00)	5.00 (2.00–10.00)	0.248
Mechanical index	0.80 (0.70–1.00)	0.80 (0.70–1.00)	0.820
Depth (cm)	8.00 (7.70–10.10)	8.00 (7.70–10.10)	0.387
Angle (°)	179.00 (178.00–180.00)	179.00 (178.00–180.00)	0.255

### Feature selection

**Fig. 4:**
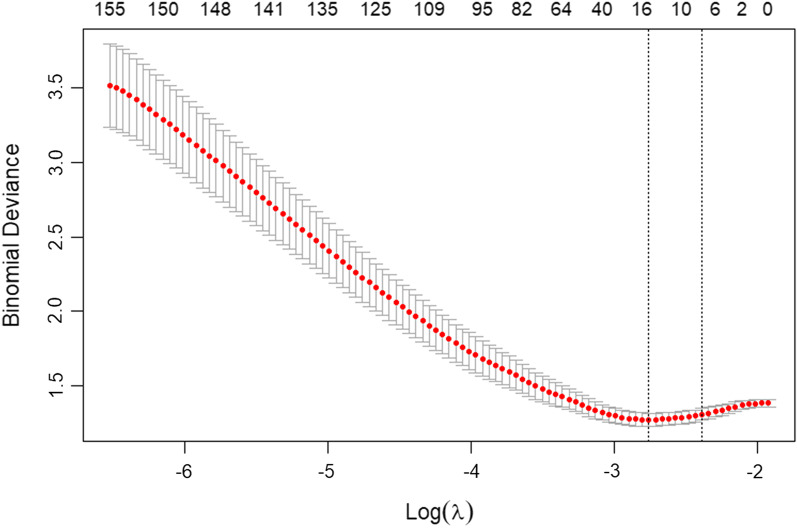
Bottom x-axes represent the value of the parameter Log (λ) of the lasso regression model, top x-axes represent the number of corresponding non-zero coefficients, and the ordinate is Binomial Deviance (binary classification anomaly), which indicates the error of the model. There are two numerical dotted lines in the picture, the line with the lowest error on the left and the line with few features on the right. We should choose the model corresponding to λ with as few variables and errors as possible. The right vertical dashed line is the best value of the logarithm, and the number of features corresponding to this value is 7

**Fig. 5:**
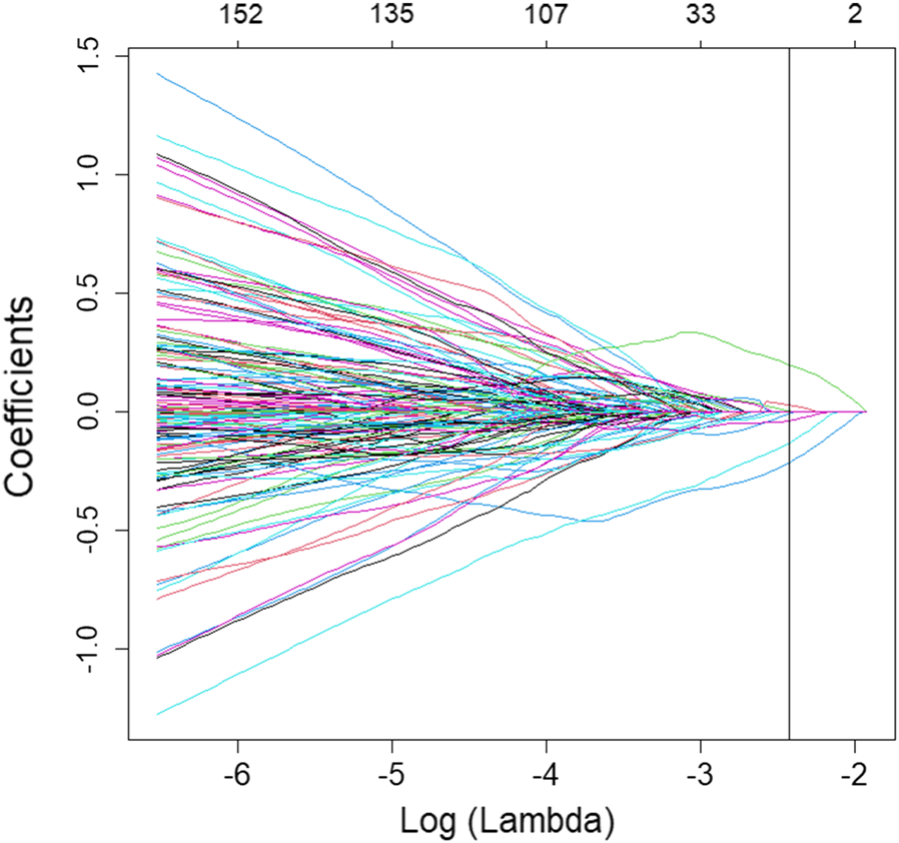
Minimum absolute contraction were used to screen the optimal features. Bottom x-axes represent the value of λ in the lasso regression model, and top x-axes represent the number of non-zero coefficients in the corresponding model at this time. With the change of the value of λ, the later the coefficient is compressed to 0, the more important it is

**Fig. 6:**
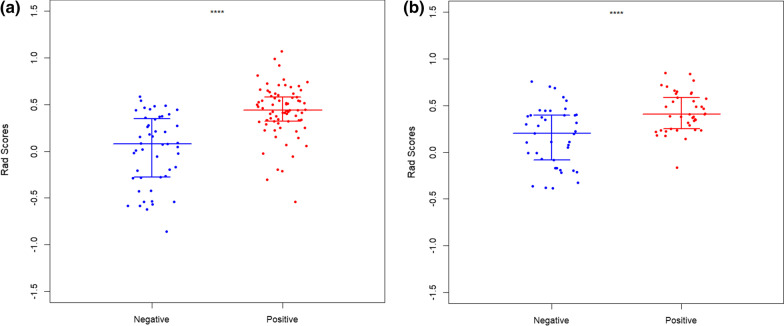
**a** show a scatter plot for train. **b** show a scatter plot for train.test. The scatter plot shows significant differences between type I and type II epithelial ovarian cancer. Negative represents type I epithelial ovarian cancer. Positive represents type II epithelial ovarian cancer

**Fig. 7:**
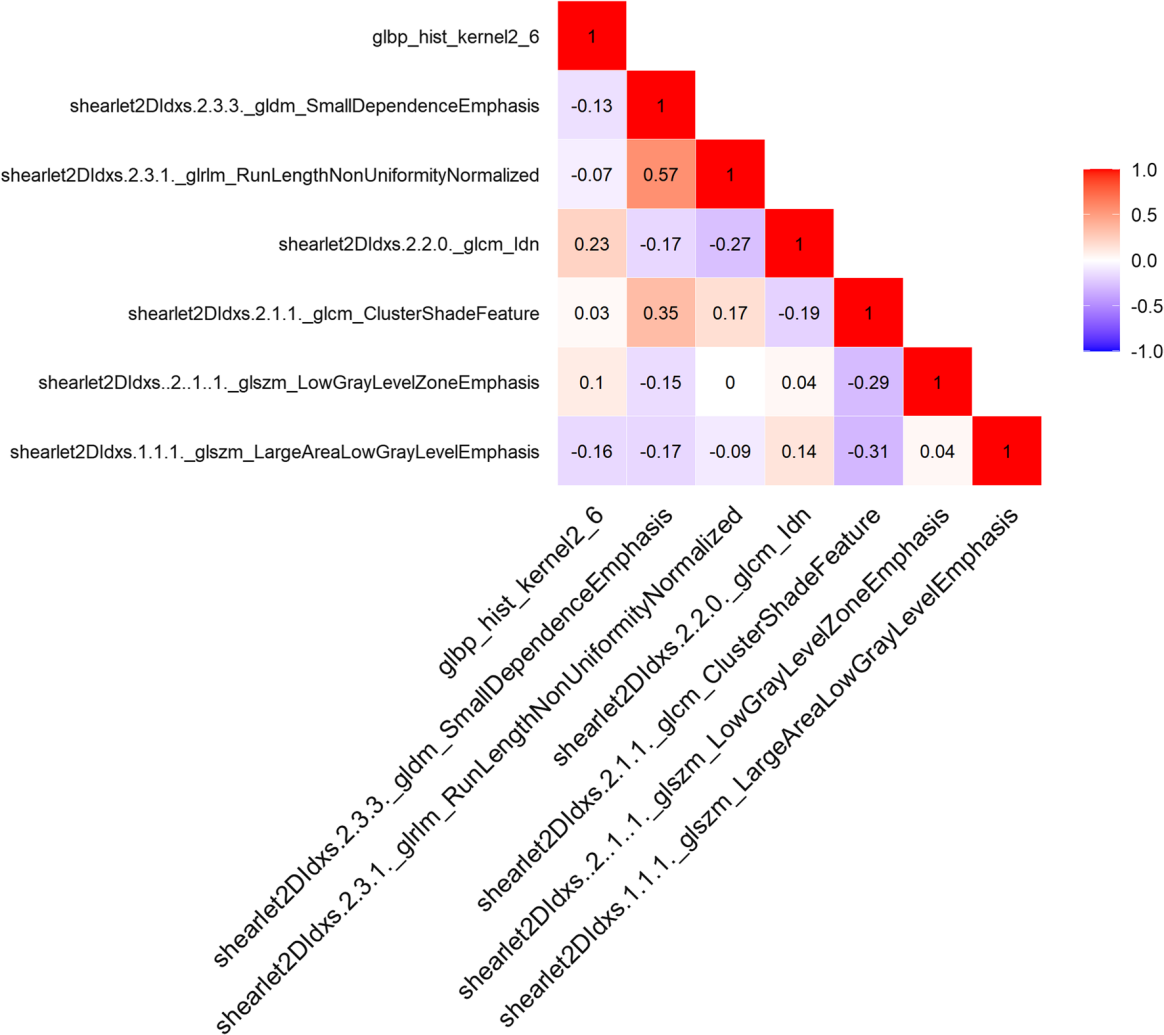
The redder the color, the higher the correlation; the bluer the color, the lower the correlation. According to the heatmap, the correlation of the seven selected features is small and the influence of multicollinearity is eliminated

In this study, a total of 4976 features were extracted from ultrasound images.To increase the comparability of quantitative radiomic features, we performed Z-score normalization for quantitative features in training and test sets. In order to reduce the influence of high-dimensional features on the model, we use lasso regression method to downscale features, and select the optimal feature subset through ten-fold cross-validation. (Figs. 4 and 5). Finally, seven features with the most differences were obtained in this study. The scatter plot showed significant differences between type I and type II epithelial ovarian cancer (Fig. 6). According to the heatmap, the correlation of the seven selected features was small, and the influence of multicollinearity was eliminated (Fig. 7).

### Image group model and evaluation

**Fig. 8:**
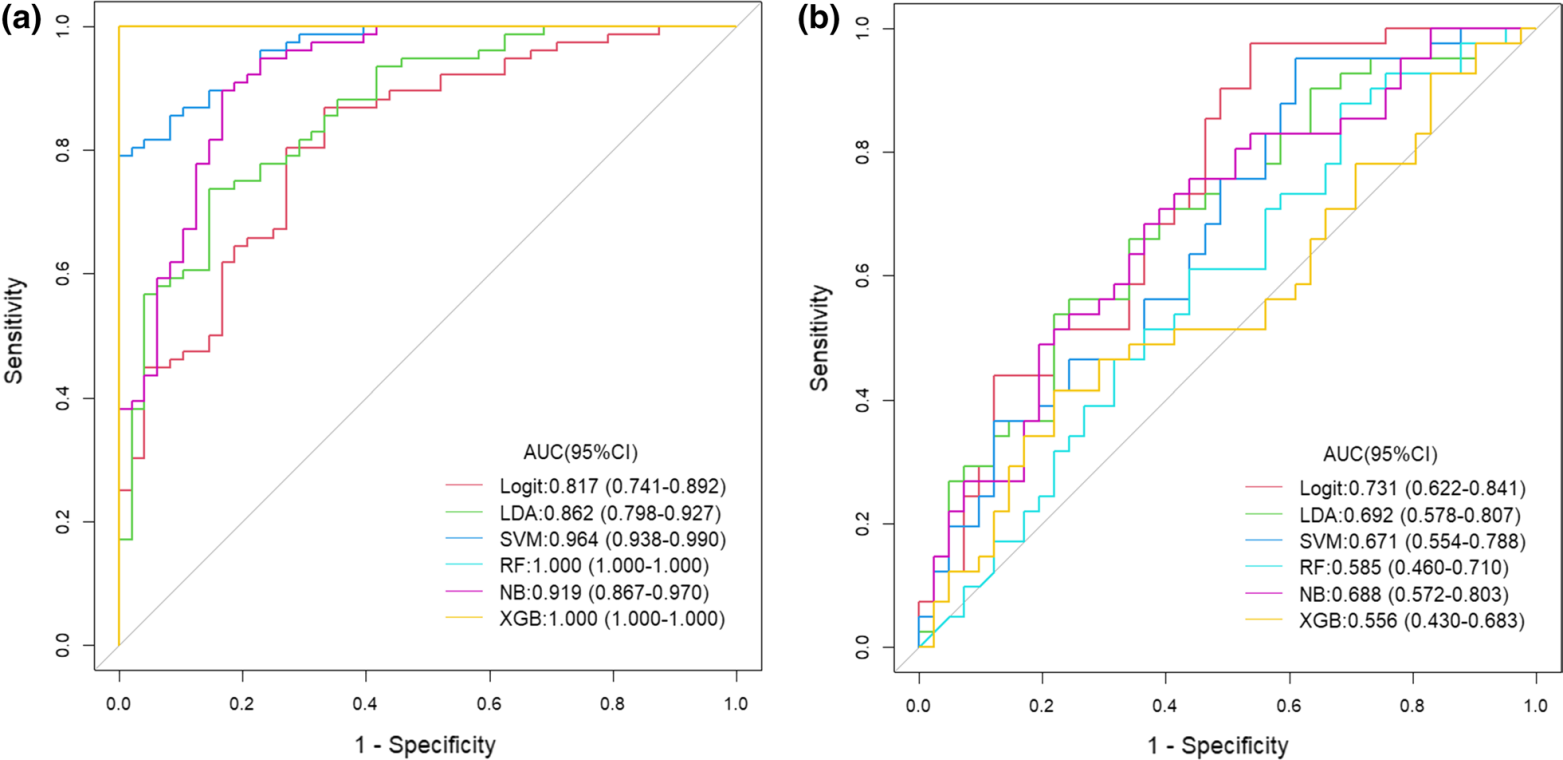
**a** show ROC for train. **b** show ROC for test. Six machine learning methods are used to construct the model (Logit: logistic regression, LDA: linear judgment analysis, SVM: support vector machine, RF: random forest, NB: naive Bayesian, XGB: limit gradient lifting algorithm)

**Fig. 9:**
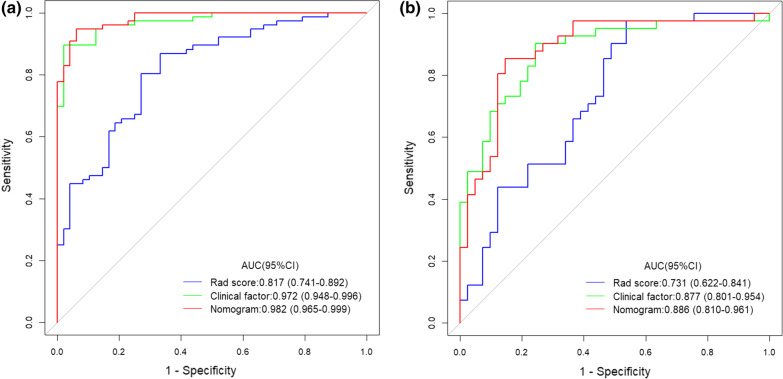
**a** show ROC for train. **b** show ROC for test. The red curve represents the radiomics model. The green curve represents the clinical model. The blue curve represents the comprehensive model. AUC, area under the receiver-operating-characteristics curve

The seven radiomics features selected were introduced into the classifier to establish a model for evaluating two different histopathological types of epithelial ovarian cancer. We used six machine learning methods (random forest, linear judgment analysis, support vector machine, logistic regression, naive Bayesian, limit gradient lifting algorithm) to construct the model learning method (Fig. 8). Finally, the method with the highest AUC value was selected, and the optimal radiomics model (logistic regression model) was established (Fig. 9). Each feature was multiplied by its regression coefficient and summed. The result was the radiomics score of each patient.

### Clinical parameter processing

Basic information (age, BMI), menopausal status, maximum tumor diameter, serum tumor marker levels, ascites and histopathological classification were collected. Serum tumor markers included carcinoembryonic antigen, glycosyl antigen 125, glycosyl antigen 153, glycosyl antigen 199, squamous cell carcinoma-associated antigen, serum human chorionic gonadotropin, human epididymal epithelial secretory protein 4, alpha-fetoprotein, the Premenopausal ROMA index and the Postmenopausal ROMA index. The above data were detected within two weeks before the operation. The markers selected by the LASSO method were analyzed by multivariate analysis, and the markers with P < 0.05 were selected to establish the prediction model.

### Comprehensive model establishment

**Fig. 10:**
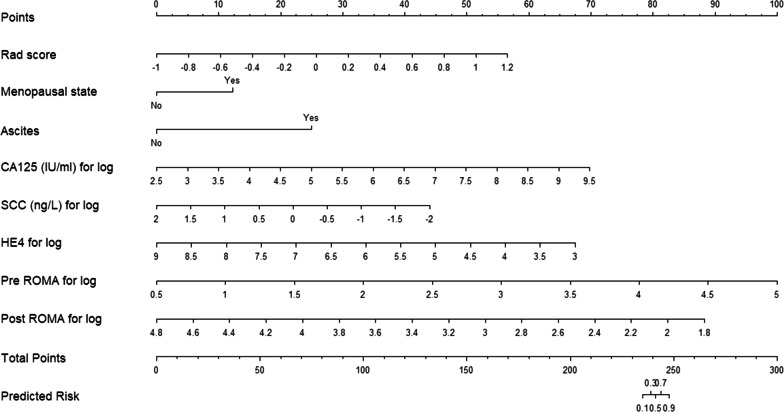
A comprehensive model nomogram is developed for the prediction of EOC type II with rad score, menopausal state,ascites,CA125,SCC,HE4,pre ROMA and post ROMA. To use this nomogram, first locate the patient’s rad score, and then draw a line straight up to the points axis on the top to obtain the score associated with rad score. Repeat the process for the other covariates (from ascites to post ROMA). Add the score of each covariate together and locate the total score on the total points axis just below the last covariate—Bovine arch axis. The values of Points corresponding to each variable were all added as the values of Total Points, and the corresponding value of Total Points is the risk value for predicting type II epithelial ovarian cancer

**Fig. 11:**
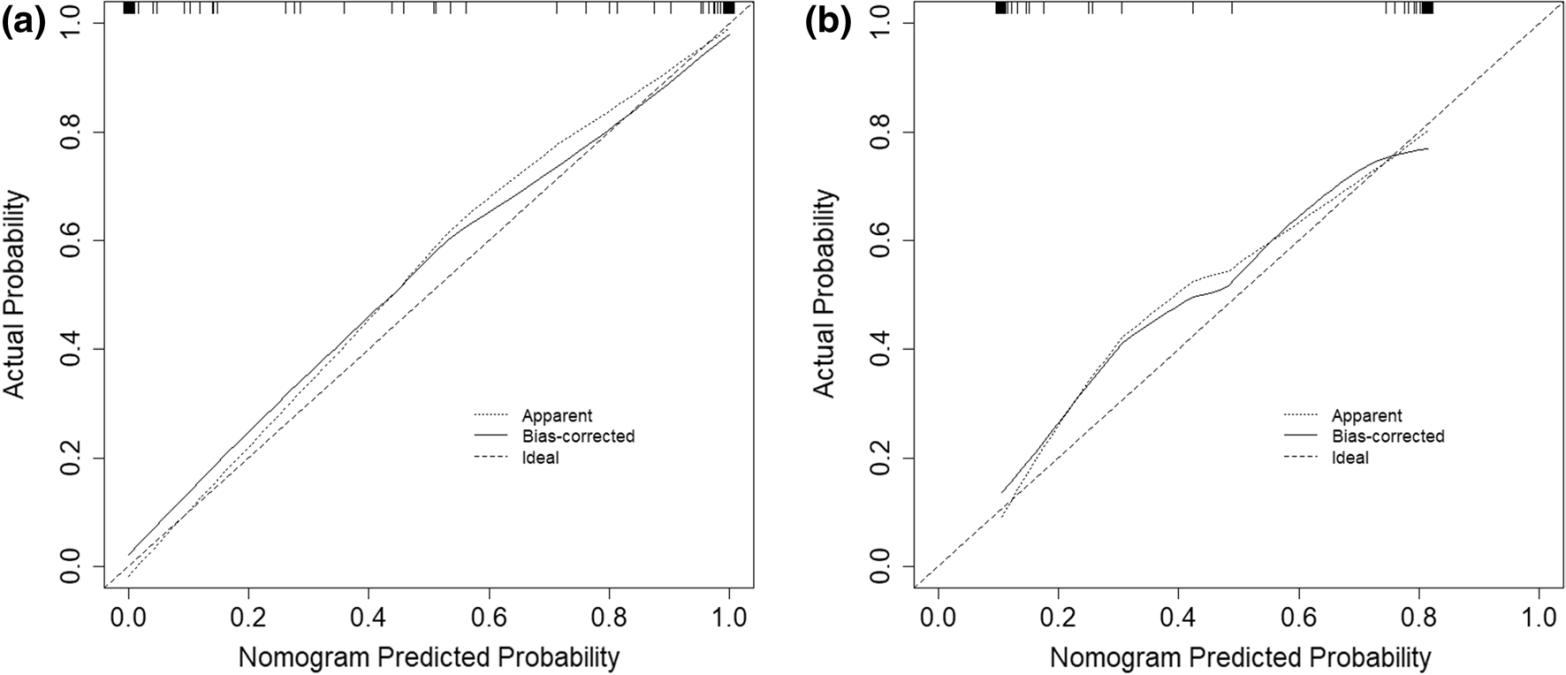
**a** show calibrate for train. **b** show calibrate for test.Calibration curves is used to evaluate the consistency of the comprehensive prediction model and the radiomics model. The results show that both models have excellent evaluation performance

**Fig. 12:**
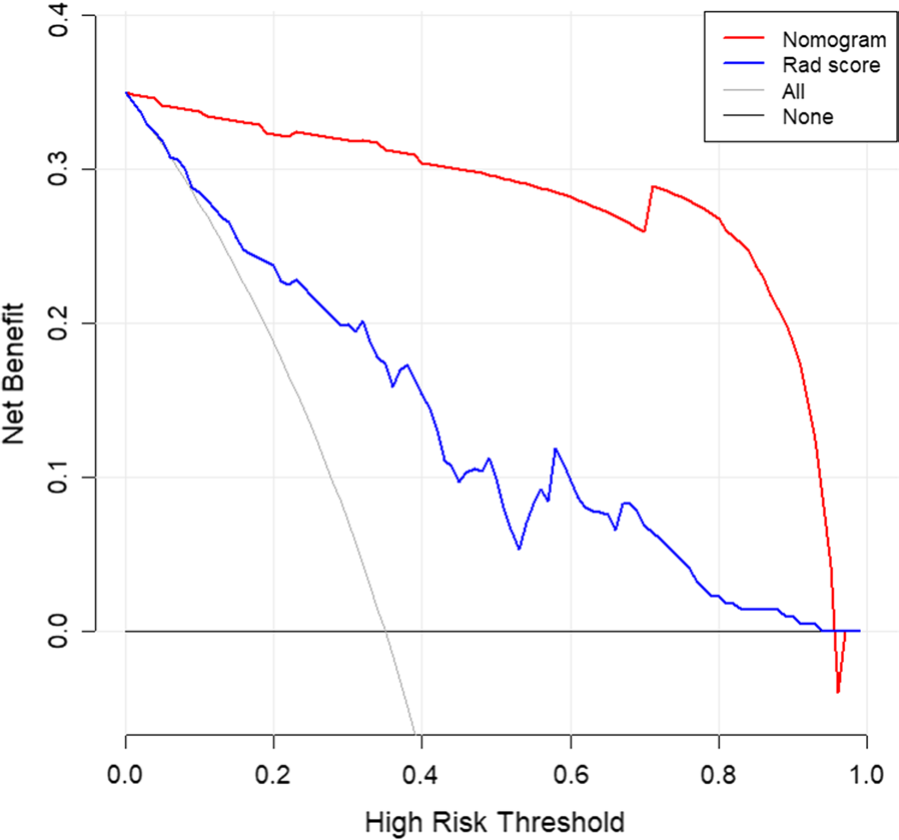
Decision curve analysis is used to assess the clinical significance of the model by quantifying the net benefits under different threshold probabilities. The blue curve represents the radiomics model, The red curve represents the comprehensive model. Both models have higher clinical benefit values

The selected features were used to construct a radiomics model by logistic regression, and a comprehensive prediction model was obtained by combining the radiomics score with clinical parameters (Fig. 9). The comprehensive prediction model is displayed in the form of nomogram (Fig. 10). Finally, the test group evaluation model was used, and the evaluation indicators were AUC, accuracy, sensitivity and specificity. Calibration curves were used to evaluate the consistency of the model (Fig. 11), and decision curve analysis (DCA) was used to assess the clinical significance of the model by quantifying the net benefits under different threshold probabilities (Fig. 12).

### Statistical analysis

R software (version 3.6.0) and *SPSS* software (version 22.0) were used for statistical analysis. For the measurement data of normal distribution, the t test of complete random design was used to compare the two samples, and the analysis of variance was used to compare several independent samples. The variables were summed as the mean ± standard deviation (*SD*). For the skewness distribution measurement data, the rank sum test was used for the progressive nonparametric test, and the variables were summed as *M* (*Q1* ~ *Q3*). The counting data were tested by the chi-square test, and the variables are presented as percentages.

## Results

### Clinical data

At the end of this study, 154 patients with epithelial ovarian cancer were enrolled, with an average age of 50.15 ± 10.80 years, ranging from 21 to 76 years old. Seventy-three patients (mean age 48.55 ± 12.17 years; range 24–76 years) were confirmed to have type I epithelial ovarian cancer by postoperative pathology; 33 of these patients had low-grade serous carcinoma, 20 had clear cell carcinoma, 9 had endometrioid carcinoma, and 11 had mucinous carcinoma. Eighty-one patients (mean age 51.36 ± 9.50 years; range 21–74 years) were confirmed to have type II epithelial ovarian cancer by postoperative pathology; 80 of these patients had high-grade serous carcinoma, and 1 of these patients had undifferentiated carcinoma. Among the 154 patients, 102 had unilateral lesions, and 52 had bilateral lesions; of the 206 total lesions, 89 were type I epithelial ovarian cancer, and 117 were type II epithelial ovarian cancer.

### Radiomics data

Among the 154 patients with epithelial ovarian cancer, the training set included 93 patients, with 62 unilateral lesions and 31 bilateral lesions. The total number of lesions in the training set was 124, and the number of type I and type II epithelial ovarian cancer lesions was 53 and 71, respectively. The test set included 61 patients, with 40 unilateral lesions and 21 bilateral lesions. The total number of lesions in the test set was 82, and the number of type I and type II epithelial ovarian cancer lesions was 36 and 46, respectively. The clinical parameters of the training set and the test set are shown in Table 2. There was no significant difference in the distribution of the clinicopathological features, including age, BMI, maximum tumor diameter, serum tumor marker levels, HE4, ROMA index, pathological subtypes, menopausal status and ascites, between the two groups.

**Table 2:** The clinical parameters of the training set and the test set are shown in

Variable	Training set (n = 124)	Test set (n = 82)	*P*
Age(years)	50.00 (43.00–56.00)	52.50 (46.00–59.00)	0.114
BMI	22.69 (20.25–25.19)	22.03 (19.92–24.20)	0.161
Maximum diameter of tumor (mm)	80.00 (53.00–105.00)	94.50 (57.00–115.00)	0.124
CEA (ng/ml)	1.70 (0.90–2.60)	1.71 (1.12–2.89)	0.431
CA125 (IU/ml)	692.00 (164.50–1384.6)	561.70 (143.50–1296.5)	0.431
CA153 (IU /ml)	34.54 (15.50–72.20)	36.35 (16.36–95.05)	0.776
CA199 (IU/ml)	12.28 (4.32–108.44)	17.32 (4.24–73.34)	0.694
SCC (ng/L)	0.70 (0.50–1.10)	0.60 (0.50–1.20)	0.830
HCG (mIU/ml)	0.56 (0.10–1.88)	1.02 (0.10–2.14)	0.149
HE4	172.70 (76.10–498.50)	159.60 (73.20–519.90)	0.602
AFP(ng/ml)	2.34 (1.79–3.27)	2.43 (1.58–3.35)	0.983
pre ROMA	69.33 (19.41–96.68)	60.97 (18.34–97.53)	0.630
post ROMA	79.61 (46.90–99.96)	76.96 (45.97–99.84)	0.502
Menopausal.state #			0.993
NO	53 (42.75)	35 (42.68)	
YES	71 (57.25)	47 (57.32)	
Ascites #			0.309
NO	45 (36.29)	36 (43.90)	
YES	79 (63.71)	46 (56.10)	
Figo stage #			0.106
I-II	50 (40.32)	24 (29.27)	
III-IV	74 (59.68)	58 (70.73)	

^#^The counting data

Pre ROMA, Premenopausal ROMA; Post ROMA, Postmenopausal ROMA

### Radiomics feature extraction

A total of 4976 features were extracted and normalized. The features with differences were screened by Lasso regression tenfold cross-validation and minimum absolute shrinkage (Figs. 4, 5). Finally, 7 optimal features were obtained. The heatmap is shown in Fig. 7. The correlation between features is represented by color. The redder the color is, the higher the correlation is. In contrast, the bluer the color is, the lower the correlation is. The heatmap shows that the correlation of the selected seven features is very small, eliminating the effect of multicollinearity. The scatter plot shows that the seven optimal features selected were quite different between type I and type II epithelial ovarian cancer (Fig. 6).

### Model construction and evaluation

The radiomics model has a high overall classification performance in identifying epithelial ovarian cancer types I and II, with AUC values of 0.817 and 0.731 in the training set and test set, respectively (Fig. 9).

Combined with the results of multivariate logistic regression analysis (Table 3), a comprehensive predictive model was constructed by combining menopausal state,ascites, CA125, SCC, HE4, Premenopausal ROMA, Postmenopausal ROMA and Radiomics score. The AUC values in the training set and test set were 0.982 and 0.886, respectively (Fig. 9). The difference in performance between the simple radiomics model and the complete nomogram is statistically significant(P < 0.05).The nomogram based on the comprehensive prediction model is shown in Fig. 10, which quantifies the factors of each patient and can be used to predict type I and type II epithelial ovarian cancer more intuitively before operation. The calibration curve shows that the radiomics model is in good agreement with the comprehensive model, as shown in Fig. 11. The decision analysis curve shows that both models have good clinical practicability, as shown in Fig. 12.

**Table 3:** The markers selected by the LASSO method are analyzed by multivariate analysis

Variable	Univariate		Multivariate	
	OR (95%CI)	*P*	OR (95%CI)	*P*
Age(years)	1.021 (0.986–1.057)	0.244	–	
Height(cm)	0.980 (0.907–1.059)	0.604	–	
Weight(kg)	0.999 (0.971–1.028)	0.955	–	
BMI	1.003 (0.933–1.077)	0.940	–	
Menopausal state	0.987 (0.976–0.999)	0.857	13.347 (2.108–84.522)	0.006
Maximum diameter of tumor (mm)	1.103 (0.938–1.298)	0.036		
Ascites	22.333 (8.599–58.007)	0.000	189.06 (17.679–2021.839)	0.000
CEA(ng/m)*	1.116 (0.761–1.636)	0.575	–	
CA125(IU/ml)*	3.621 (2.315–5.663)	0.000	10.357 (3.543–30.275)	0.000
CA153(IU/ml)*	1.976 (1.342–2.908)	0.001	–	
CA199(IU/ml)*	0.939 (0.782–1.128)	0.504	–	
SCC(ng/L)*	0.767 (0.4–1.468)	0.422	0.131 (0.032–0.528)	0.004
HCG(mIU/ml.)*	0.928 (0.796–1.081)	0.338	–	
HE4*	1.278 (0.954–1.711)	0.100	0.262 (0.097–0.709)	0.008
AFP(ng/ml)*	1.563 (0.868–2.814)	0.137	–	
Pre ROMA*	1.602 (1.089–2.358)	0.017	20.908 (2.6–168.14)	0.004
Post ROMA*	2.281 (1.163–4.474)	0.016	0.001 (0–0.097)	0.003

*For log

Pre ROMA, Premenopausal ROMA; Post ROMA, Postmenopausal ROMA

## Discussion

The ultrasound images of 154 patients with epithelial ovarian cancer were included in this study. The quantitative expression values of imaging features were normalized with the Z-score method, and the 7 most different features were screened by using the Lasso regression tenfold cross-validation method. Then, six machine learning methods (random forest, linear judgment analysis, support vector machine, logistic regression, naive Bayesian, limit gradient lifting algorithm) were used to construct the model, and the method with the highest AUC value was selected to establish the logistic regression radiology model. The AUCs of the training group and test group were 0.817 and 0.731, respectively. In addition, combined with the clinical indexes of the patients, the data regarding serum tumor markers monitored two weeks before operation were collected and analyzed by multivariate logistic regression analysis. Finally, menopausal state,ascites, CA125, SCC, HE4 and Premenopausal and Postmenopausal ROMA were identified as independent factors. The above indexes were combined with the radiomics score to create a comprehensive prediction model, and the AUCs in the training group and test group were 0.982 and 0.886, respectively. The nomogram of this study quantifies the value of each factor of the patient and visually shows the efficiency of the comprehensive model in predicting two kinds of epithelial ovarian cancer. From the value of AUC, the efficacy of comprehensive prediction model is higher than radiomics model. After analysis, the following factors were obtained. Firstly, this study collected a lot of clinical indicators, including maximum tumor diameter,ascites,carcinoembryonic antigen, glycosyl antigen 125, glycosyl antigen 153, glycosyl antigen 199, squamous cell carcinoma-associated antigen, serum human chorionic gonadotropin, human epididymal epithelial secretory protein 4, alpha-fetoprotein, the Premenopausal ROMA index and the Postmenopausal ROMA index. The two indexes of maximum tumor diameter,ascites came from ultrasonic testing. Therefore, these indexes are not only simple blood test indexes, but also comprehensive indexes of patients' basic information, blood biochemistry and ultrasonic examination. Ultrasound has certain advantages in daily work.Ultrasonic operation is convenient, simple and non-invasive. It can also monitor the size of the mass and the presence of ascites. Therefore, ultrasound is a common method to detect ovarian tumors in clinical work. At the same time, radiomics also provides a new evaluation method for clinic, which has a certain potential value. In this study, the clinical decision curve shows that both the imaging model and the joint model are located above the None line and the All line, indicating that both models are valuable in predicting the classification of epithelial ovarian cancer, and the net return of the combined model is higher than that of the radiomics model. Because the progression of type I and type II epithelial ovarian cancer is different, it is of clinical significance to correctly predict the two types.

Radiomics, first proposed by Lambin et al. in 2012 [11], has developed rapidly in recent years [13]. It provides a noninvasive method for diagnosing and predicting diseases and is widely regarded as a step in the development of radiomics for personalized cancer management. At present, most studies are based on CT and MR images. Qian et al. retrospectively analyzed 65 patients with epithelial ovarian cancer using conventional MRI images to compare the differences between radiomics models and traditional models in identifying early and advanced epithelial ovarian cancer [22]. Zhu et al. performed a retrospective analysis of 101 patients with ovarian cancer based on CT images and established a model to distinguish epithelial ovarian cancer from nonepithelial ovarian cancer by radiology combined with a clinical model [23]. Compared with the above studies, the purpose of our study was more precise, specifically for the preoperative prediction of pathological subtypes of epithelial ovarian cancer, which may provide a new approach for the current accurate medical treatment. In addition, our study had more patients and features, including a total of 154 patients and extracting 4976 radiomic features. Compared with CT and MRI, ultrasound has the advantages of being simple operate and providing real-time observation, and it plays an important role in the diagnosis and treatment of ovarian tumors [24–26]. Therefore, with the remarkable progress of computer technology, the attempt to apply artificial intelligence to clinical practice has become increasingly feasible, and there will certainly be important breakthroughs in the future [13, 27–30].

However, our research also has some limitations. First, all the ultrasound imaging data came from a single center, and the study was retrospective, so it was necessary to conduct a multicenter prospective study. Second, our study only included epithelial ovarian cancer, excluding benign, borderline, sex cord interstitial and germ cell tumors of the ovary. We will add data on other types of ovarian tumors in future studies to optimize the universality and clinical value of the model. In addition,We are also trying to use radiomics to distinguish benign and malignant ovarian tumors, hoping that it can provide us with new ideas in differential diagnosis.

## Conclusions

In summary, we developed and validated a radiomics model based on ultrasound to distinguish different histopathological types of epithelial ovarian cancer. Thus, it provides clinicians with a new method for the noninvasive preoperative identification of type I and type II epithelial ovarian cancer.

## Data Availability

The datasets generated and analyzed during the current study are not publicly available due to the original datasets containing personal privacy information but are available from the corresponding author on reasonable request.
